# Therapy-Related Myeloid Neoplasm in Non-Hodgkin Lymphoma Survivors

**DOI:** 10.4084/MJHID.2011.065

**Published:** 2011-12-20

**Authors:** Alessia Bari, Luigi Marcheselli, Raffaella Marcheselli, Eliana Valentina Liardo, Samantha Pozzi, Paola Ferri, Stefano Sacchi

**Affiliations:** 1 Program of Innovative Therapy in Oncology and Hematology, Department of Oncology and Hematology, University of Modena and Reggio Emilia, Modena, Italy; 2 Department of Public Health Sciences, University of Modena and Reggio Emilia, Modena, Italy

## Abstract

Relatively little data on secondary cancers is available regarding patients treated for non-Hodgkin lymphoma (NHL), compared with those treated for Hodgkin lymphoma. Evolving treatment regimens have improved survival outcomes for NHL patients. As a result of this improvement, secondary malignancies are becoming an important issue in NHL survivors. This review aims to report data on this topic previously published by our group, adding unpublished results from the Modena Cancer Registry (MCR). We recently performed four studies about secondary neoplasms in NHL survivors: two studies analysing the risk of secondary neoplasms in patients treated for indolent and aggressive NHL; a meta-analysis of 23 studies investigating the risk of secondary malignant neoplasm (SMN) after NHL treatment; and a still-unpublished study evaluating the incidence of therapy-related myeloid neoplasm (t-MN) in patients treated for NHL (from the MCR database). The first two studies analysed 563 patients with indolent NHL and 1280 patients with diffuse large B-cell lymphoma (DLBCL) enrolled in the Gruppo Italiano Studio Linfomi (GISL) trials. Results showed that the cumulative incidence of secondary tumours was 10.5% at 12 years for indolent NHL and 8.2% at 15 years for DLBCL. Results of the meta-analysis indicated that NHL patients experienced a 1.88-fold increased risk for SMN compared with the general population; the standardized incidence risk (SIR) for secondary acute myeloid leukaemia (AML) was 11.07. Based on data from the MCR from 2000 through 2008, we found that the SIR was 1.63 for developing a secondary malignancy after NHL, and 1.99 for developing secondary haematological malignancies. Regarding myelodysplastic syndrome and/or AML incidence, nine NHL patients developed t-MN with a higher risk than expected (SIR 8.8, 95% CI: 4.0–16.6). In conclusion, patients treated for NHL are at increased risk of developing SMN. Regarding t-MN, data from the meta-analysis and the MCR demonstrate an excessive risk of developing AML (SIR 11.07 and 5.7, respectively) compared with solid SMN after treatment for NHL. Thus long-term monitoring should be considered for NHL survivors.

## Introduction

Improved survival outcomes for non-Hodgkin lymphoma (NHL) patients, particularly due to the introduction of monoclonal antibodies^1^–^4^ in combination with chemotherapy, have raised the issue of late treatment sequelae such as secondary tumours. Several^5^–^10^ but not all^11^–^13^ studies have reported an increased risk of developing secondary cancers in NHL survivors; however, few publications contain clinical characteristics and therapy data that are useful for identifying risk factors for the development of secondary malignancies related to lymphoma treatment. This lack is partly because many studies have analysed data from population-based registries, which usually do not provide information about histology subset or therapeutic approaches.^6^,^7^,^9^–^11^

In this review, which includes some unpublished results based on data from the Modena Cancer Registry (MCR), we focus on therapy-related cancers, including myeloid neoplasms, observed in NHL patients enrolled in the Gruppo Italiano Studio Linfomi (GISL) trials, and a meta-analysis that we performed on 23 studies published on this topic.^14^

In two previously published studies, our group analysed two homogeneous groups of patients with indolent^15^ and aggressive^16^ NHL treated at GISL centres to determine the incidence rate and risk factors for secondary cancers, particularly therapy-related myelodysplastic syndromes/acute myeloid leukemia (MDS/AML). In addition, we have recently performed a meta-analysis to estimate the pooled relative risk (RR) of secondary malignant neoplasm (SMN) in NHL survivors^14^ and the association between chemotherapeutic or radiotherapeutic approaches and site-specific cancers, focusing our attention on MDS and AML. Finally, we compared meta-analysis results with those obtained from MCR data. This publication aims to provide a broad overview of incidence and risk factors for therapy-related secondary neoplasia, a condition that is frequently addressed, but never deeply analysed with specific investigations.

## Design and Methods

Data regarding secondary malignancies in patients with indolent lymphoma (follicular, marginal zone, and small lymphocytic lymphomas)^15^ or aggressive lymphoma (diffuse large B-cell lymphoma, DLBCL)^16^ treated between 1988 and 2003 have been extracted from the GISL database, located in Modena, Italy. The GISL registry collects clinical information and treatment schedules of all GISL clinical trials from enrolment to follow-up. Information is updated every 3–6 months during the study period, and every 12 months during the follow-up. The inclusion criteria and statistical method used are reported in the original papers.^15^,^16^ Among 625 indolent lymphomas enrolled in several clinical trials,^17^–^23^ we identified a total of 563 patients who met all inclusion criteria. In the second study, 1280 patients among 1387 cases with DLBCL were selected and evaluated for secondary neoplasm. The main goals of our studies were to determine the percentage of SMN in our cohort, the standardized incidence ratio (SIR), and the risk factors for developing secondary cancer in lymphoma-treated survivors.

The meta-analysis^14^ was performed by reviewing papers about secondary neoplasia selected from electronic databases (Medline and Embase) to provide a global quantitative assessment of the risk for SMN. Search strategy, selection criteria, data extraction, and statistical analysis are extensively described in the original article. Every effort to avoid selection bias was adopted. A total of 1,521 citations were identified from the electronic search; at the end of selection, 23 papers satisfied all inclusion criteria.

The unpublished results that we describe herein come from the MCR database. The analysis was addressed to the identification of secondary cancer after primary neoplasm, particularly after NHL, diagnosed between 2000 and 2008. Data from the MCR allows us to evaluate incidence and RR of SMN, and particularly of therapy-related myeloid neoplasms (t-MN), from a database population registry. These data reflect clinical practice in a well-defined time period and geographical area.

## Results

### Clinical trial results

In the study on secondary cancer after indolent lymphoma,^15^ 39 patients out of 563 (6.9%) developed an SMN after a median follow-up of 62 months. We observed 12 MDS/AML cases, with a median time between the diagnosis of lymphoma and t-MN of 25 months (range 6–168 months), highlighting the onset of myeloid neoplasm within 2–3 years after the first tumour. We also identified 27 solid cancers, with a median time from diagnosis of NHL to diagnosis of SMN of 52 months (range 16–164 months). In the study population, the risk of a secondary tumour was higher (SIR 1.9, 95% CI: 1.4–2.7) than the risk of malignancy in the general Italian population, particularly among male patients (SIR 2.72; 95% CI: 1.76–4.02, *p*=0.016) and in patients younger than 65 years (SIR 2.66; 95% CI: 1.69–4.0, *p*=0.037) (Table 1). In particular, SIR was 4.91 (95% CI: 2.2–10.1) in the cohort groups aged 45–54 years, and 3.41 (95% CI: 1.98–5.87) in those aged 55–64 years. Regarding treatment, we observed an increased risk for each schedule of therapy. In particular, fludarabine-containing regimens were associated with a higher risk of developing a secondary neoplasm (SIR 3.41; 95% CI: 1.81–5.83). It should be noted that we calculated the overall SIR of secondary malignancies excluding MDS/AML cases, as the incidence rates of these malignancies are not reported by the Italian Institute of Health. In univariate and multivariate analysis, age, male sex, and fludarabine-based treatment each had a negative impact on time free from secondary tumours.

In our second study, which focused on the evaluation of SMN after DLBCL^16^ in patients treated in GISL trials,^24^–^34^ 48 patients (3.8%, crude rate 7.6 per 1000 person-years) out of 1280 developed a second cancer. Of these 48 patients with secondary cancers, eight developed MDS (n=5) or AML (n=3), five developed other haematological malignancies, and 35 developed solid tumours. Fourteen out of 48 secondary malignancies occurred after additional treatments for progressive or recurrent disease. The median time between the diagnosis of DLBCL and secondary solid tumour diagnosis was 71 months (range, 13–176 months); for patients who developed secondary MDS/AML, the median time between diagnosis of DLBCL and t-MN was 43 months (range 30–127 months). In contrast to patients with indolent NHL, the overall risk of secondary cancer in patients with DLBCL was similar to that observed in the general Italian population (SIR 1.1; 95% CI: 0.8–1.5) (Table 2). We did not calculate the SIR of MDS/AML, as the incidence rates of these malignancies are not reported by the Italian Institute of Health. Sex, international prognostic index score, chemotherapy regimens, radiotherapy, number of chemotherapy lines, and time of first treatment did not have any significant impact on the SIR of developing a secondary cancer. However, an increased and statistically significant risk of SMN was observed in the cohort groups of 20–39 years and 40–59 years of age. The cumulative incidence of SMN after correction in a competing-risk model was 2.3%, 4.7%, and 8.2% at 5, 10, and 15 years, respectively. Considered separately, the cumulative incidence at the same time intervals was 1.5, 3.3, and 6.8 for solid tumours, and 0.8, 1.4, and 1.4 for haematological malignancies, respectively. We did not observe any plateau in the curve of diagnosis of secondary solid tumours, while the curve of haematological malignancies stopped increasing after 10 years. The only factor that had a significant negative impact on the development of secondary tumours was age >60 years at first treatment; no other factor appeared to significantly influence the development of SMN. In a separate analysis, no variable appeared to be associated with the development of MDS/AML.

### Meta-analysis results

**Figure 1:**
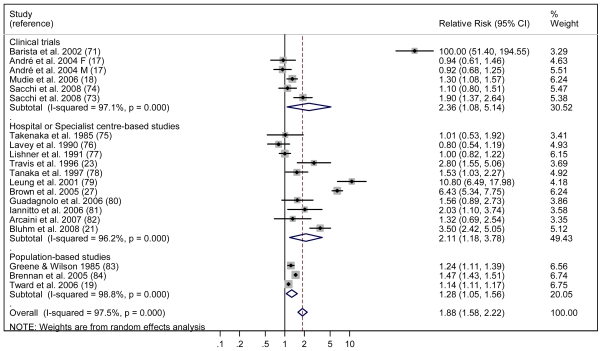
Forest plot of the meta-analysis relating risk for secondary solid tumors in NHL survivors.

**Figure 2:**
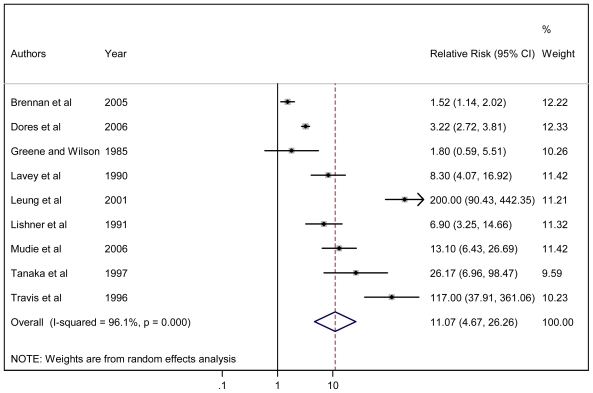
Forest plot of the meta-analysis relating risk for secondary AML in NHL survivors

For the meta-analysis,^14^ we initially identified 1,521 potentially eligible studies. Based on the inclusion criteria, 23 papers were analysed; of these, 21 studies contributed to principal meta-analysis on the risk of developing SMN, and 19 provided risk factors for specific cancer types. For the evaluation of overall secondary malignancy risk, we analysed 23 studies that included a total of 208,643 NHL survivors who developed 13,878 SMN recruited during the period 1935–2004. The pooled RR of SMN was 1.88 (95% CI: 1.58–2.22), an increased, statistically significant value in comparison with the risk of the general population (Figure 1). When separately calculated, the pooled RRs for clinical trials, hospital-based studies, and population-based studies were 2.36, 2.11, and 1.27, respectively. To evaluate the risk of secondary AML, 19 studies including a total of 197,456 NHL survivors recruited during the period 1935–2004 were reviewed. The calculated RR for AML in this population was 11.07 (95% CI: 4.67–26.26) (Figure 2). For all studies, meta-analysis revealed a significant association between previous NHL and the risk of developing secondary solid cancers with a RR of 1.32 (95% CI: 1.07–1.63), which was extremely different from the RR for AML. Younger age and exposure to total body irradiation were significantly associated with an increased risk of developing a secondary tumour.

The use of any type of chemotherapy alone was associated with a higher risk of developing a SMN. A similar result was observed in the sub-analysis of patients treated with alkylating agents only; while the pooled RR of SMN for patients that received treatment with cyclophosphamide, adriamycin, vincristine, and prednisone (CHOP) or CHOP-like therapy or radiotherapy alone was increased, but not to the point of statistical significance. The combination of chemotherapy and radiotherapy was significantly associated with an increased risk of overall SMN, but not of solid tumours.

### Modena Cancer Registry results

From the review of the MCR database between 1990 and 2008, we found 332 cases with double diagnoses of myeloid malignancies associated with any other type of tumour. Of these, 148 were chronic myeloproliferative disorders and were excluded from the analysis. The remaining 184 cases had a diagnosis of leukemia/MDS; of these, 51 were MDS diagnosed before another cancer and 133 were leukemia/MDS diagnosed after other tumours. Notably, more than 50% of these 133 cases were diagnosed after breast, gastrointestinal, and urothelial cancers.

Like other population-based registries, the MCR does not record data about clinical features, molecular markers, cytogenetics, treatments, etc. The collection of these data (called high resolution analysis) is actually ongoing in a separate database.

Between 2000 and 2008 in the province of Modena, the SIR for SMN after NHL was 1.63 (95% CI: 1.44–1.85), and the SIR for secondary haematological neoplasm was 1.99 (95% CI: 1.27–3.12). In this population, nine NHL patients developed t-MN (SIR 8.8; 95% CI: 4.0–16.6); based on these findings, the SIR was 5.7 (95% CI: 1.2–16.7) for AML, and 6.7 (95% CI: 2.4–14.5) for MDS.

## Conclusions

Today, successful treatments have improved the life expectancy of patients with NHL, and the risk of late treatment effects is becoming an important concern. Although the majority of published studies have shown that NHL patients are at a greater risk of developing secondary malignancies, the results of some studies are conflicting. In this article, we review data from three studies published by our group reporting risks of post-treatment secondary neoplasia in patients with indolent^15^ and aggressive NHL^16^ enrolled in GISL clinical trials, and the results of a meta-analysis of 23 publications from 1985 to 2008.^14^ We also report previously unpublished data from the MCR about the risk of developing a secondary tumour in NHL survivors. While the first two studies evaluate the risk in selected patients enrolled in clinical trials, the data from the MCR and the meta-analysis refer to risk in a non-selected population, therefore providing findings that more closely resemble clinical practice.

The two studies on survivors of indolent^15^ and aggressive NHL^16^ analysed large and homogeneous cohorts of patients who participated in clinical trials at GISL centres for a time period spanning more than 15 years. Patients’ data were recorded prospectively in the GISL database; however, the results must be interpreted cautiously because of the retrospective nature of the analysis, that may underestimate the risk of secondary cancer.

Looking at the results in patients treated for indolent NHL, the overall risk of SMN, excluding MDS/AML, appeared only slightly increased compared with the risk of malignancy observed in the general population. Increased risk of malignancy was observed in males and in patients aged 45–64 years; the risk in patients older than 65 was equivalent to the risk of cancer in the general population. The overall RR of secondary malignancy was increased for each treatment combination, but was particularly elevated among patients treated with fludarabine-based chemotherapy. The possible role of fludarabine as a risk factor for secondary t-MDS/AML has been recently reported in patients treated for chronic lymphocytic leukemia and indolent lymphoma.^35^ In conclusion, young age at the time of diagnosis, male sex, and fludarabine-based treatment emerged as negative risk factors for SMN. Considered together with conventional prognostic factors and the possible side effects of treatment, these data could be a helpful device to support physicians in choosing the most appropriate therapy.

In the cohort of patients treated for DLBCL, our results demonstrated that the overall incidence of secondary malignancies was not significantly increased. However, the risk of developing a secondary cancer was clearly age-related (as in survivors of indolent lymphoma): a strongly increased risk was observed in young patients, while the incidence in patients aged ≥60 years was equivalent to the incidence in the general population. Clinical characteristics, treatment regimen, and being treated with radiotherapy after chemotherapy did not have any influence over the SIR. Use of the new approach of involved field radiotherapy or a short follow-up period may be possible reasons for these findings.

Our observations of the cumulative incidence of secondary tumours for survivors of DLBCL were similar to those reported by other authors. However, while we did not observe any plateau in the curve of diagnosis of secondary solid neoplasia (cumulative incidence was still increasing after 12–15 years), the cumulative incidence of hematologic malignancies stopped increasing after 10 years. A longer follow-up is needed to confirm that the risk in our cohort is the same as that in the normal population. Taking into account the possible occurrence of secondary neoplasia, long-term follow-up should be considered.

The meta-analysis was conducted to estimate the risk of SMN for patients with previous NHL in a wide population. In this extensive research, we observed that NHL survivors have approximately twice the increased risk of SMN compared with the general population. Limiting the analysis to solid cancers, we also observed an increased risk associated with younger age, as in our previous study of patients with NHL. The possible association between treatment exposure and SMN risk was not explored in detail because treatment information was limited for some studies. In any case, we observed that the increased risk of secondary cancer was related to the degree of exposure to alkylating agents, alone or in combination with radiotherapy. A stronger association was observed for patients undergoing total body irradiation, although the correlation between radiation therapy and SMN has not been completely clarified. A recent publication reported an increased risk of secondary solid tumours, but not secondary MDS/AML, in patients receiving radiotherapy after high dose treatment for lymphoma.^36^ We know that our meta-analysis has specific limitations (e.g. lack of data from unpublished studies, restriction to English-language publications). However, the likelihood of publication bias in our results is small and not statistically significant. In addition, there is a large degree of variability among the selected papers regarding characteristics such as study design, NHL histology, year of recruitment, follow-up duration, type of treatment administered, and environmental and genetic influences. In particular, the studies cover an extended period of time (1935–2004) during which great changes occurred in therapeutic regimens, resulting in a significant change in responses to therapy. Strengths of this study include the use of rigorous systematic review and meta-analysis techniques to retrieve and analyse data.

Finally, data from the MCR demonstrated similar results: an increased risk of SMN and t-MN in NHL survivors, with comparable results in population-based studies and in clinical trials. High-resolution analysis is ongoing to define the clinical features and chemotherapy in detail.

In conclusion, these results confirm that NHL survivors experience a higher risk of developing SMN than the general population, and highlight the variety of possible carcinogenic effects of different chemotherapeutic approaches and combined-modality therapies, particularly in younger patients. Regarding t-MN, data from the meta-analysis and the MCR demonstrate an excessive risk of developing AML (SIR 11.07 and 5.7, respectively) compared with solid SMN after treatment for NHL. Certainly, additional analysis and longer follow-up periods are needed to confirm these observations.

## Figures and Tables

**Table 1:** Standardized Incidence Risk (SIR) according to demographics and treatment in indolent lymphoma survivors.

*Factor*	*SIR (95%CI)*	*p value*
***Overall SIR***	***1.9*** *(1.4–2.7)*	
**Gender**		
F	**1.10** (0.50–2.10)	0.016
M	**2.72** (1.76–4.02)	
**Age at first treatment**		
<65	**2.66** (1.69–4.00)	0.037
65+	**1.26** (0.63–2.26)	
**Histology**		
FL	**2.58** (1.58–3.98)	0.251
SLL	**1.49** (0.80–2.55)	
MZL	**1.15** (0.03–6.43)	
**RT-IF**		
No	**1.88** (1.25–2.71)	>0.5
Yes	**2.48** (0.91–5.40)	
**Chemotherapy containing**		
Alk	**1.54** (0.74–2.83)	0.074
Alk+Anthra	**1.57** (0.78–2.80)	
Alk+Anthra+Fluda	**3.41** (1.81–5.83)	

FL: follicular lymphoma; SLL: small lymphocytic lymphoma; MZL: marginal zone lymphoma; Alk: alkylating agent; Anthra: anthracycline; Fluda: fludarabine.

**Table 2:** Standardized Incidence Risk (SIR) according to demographics and treatment in DLBCL survivors.

Factor	SIR (95%CI)	p value
***Overall SIR***	***1.1****(0.8–1.5)*	
**Gender**		
F	**1.00** (0.57–1.62)	0.618
M	**2.72** (1.76–4.02)	
***IPI***		
0–1	**1.13** (0.69–1.75)	0.959
02-mag	**1.15** (0.71–1.76)	
**Chemotherapy**		
PCB-epidoxorubicin	**1.22** (0.78–1.81)	0.610
PCB-idarubicin	**1.12** (0.45–2.31)	
PCB-sequential	**1.24** (0.46–2.70)	
CHOP or CHOP like	**0.60** (0.16–1.55)	
**RT-IF**		
No	**1.16** (0.79–1.63)	0.549
Yes	**0.92** (0.42–1.75)	
**Cohort Age**		
20–39	**23.0** (5.76–92.0)	
40–59	**4.39** (1.92–1.78)	

PCB-epidoxorubicin: ProMECE-CytaBOM: (methylprednisolone, cyclophosphamide, epidoxorubicin -or doxorubicin-, etoposide, cytarabine, bleomycin, vincristine, methotrexate); PCB-idarubicin: ProMICE-CytaBOM (methylprednisolone, cyclophosphamide, idarubicin, etoposide, cytarabine, bleomycin, vincristine, methotrexate); PCB-Sequential: sequential ProMECE instead of the classical cycling regimen; CHOP: cyclophosphamide, doxorubicin vincristine, prednisolone. RT-IF: radiotherapy-involved field.
